# Menopausal Hormone Therapy Decreases the Odds of Developing Soft‐Tissue Disorders of the Shoulder in Perimenopausal Women

**DOI:** 10.1002/ars2.70158

**Published:** 2026-11-08

**Authors:** Isha K. Shah, Aakash K. Shah, Robert J. Burkhart, Caroline A. Dannacher, Natasha N. Desai, Jacob G. Calcei, Abigail L. Campbell

**Affiliations:** ^1^ Case Western Reserve University School of Medicine Cleveland Ohio U.S.A.; ^2^ Keck School of Medicine Los Angeles California U.S.A.; ^3^ University Hospitals Drusinsky Sports Medicine Institute Cleveland Ohio U.S.A.; ^4^ NYU Langone Health New York New York U.S.A.

## Abstract

**Purpose:**

To determine whether perimenopausal women receiving menopausal hormone therapy (MHT) have decreased odds of developing soft‐tissue shoulder disorders compared with women without an active MHT prescription, specifically adhesive capsulitis, bursitis, bicipital tendinitis, calcific tendinitis, atraumatic rotator cuff tear/rupture, and shoulder impingement syndrome and to quantify the impact of differing formulations and administration route.

**Methods:**

The TriNetX US Collaborative Research Network, a deidentified database of over 180 million patients, was queried on June 11, 2026. Women aged 40 to 60 years in a menopausal state were identified by International Classification of Diseases code N95.1 and primary ovarian insufficiency by International Classification of Diseases code E28.3. Patients were grouped by MHT prescription status. Patients were one‐to‐one propensity‐matched on age, race, ethnicity, and metabolic disease status, with standardized mean difference less than 0.1. The odds of developing soft‐tissue shoulder disorders were studied, with *P* values less than .05 considered statistically significant.

**Results:**

A total of 296,992 patients were included. The index the mean age was 49.1 years, 74.5% to 74.6% were White, 6% were Hispanic/Latino, 9% were African American, and 4.1% were Asian. Women prescribed any form of systemic MHT showed significantly lower odds of developing adhesive capsulitis (odds ratio [OR] 0.875, 95% confidence interval [CI] 0.786‐0.973; *P* = .014), bicipital tendinitis (OR 0.775, 95% CI 0.669‐0.898; *P* = .001), calcific tendinitis (OR 0.829, 95% CI 0.706‐0.974; *P* = .023), shoulder impingement syndrome (OR 0.873, 95% CI 0.792‐0.962; *P* = .006), and rotator cuff tear/rupture (OR 0.892, 95% CI 0.804‐0.991; *P* = .032). Topical estrogen specifically was associated with decreased odds of developing adhesive capsulitis, bursitis, bicipital tendinitis, shoulder impingement syndrome, and rotator cuff tear/rupture.

**Conclusions:**

In peri‐ and postmenopausal women, MHT use was associated with decreased odds of soft‐tissue shoulder disorders compared with individuals not using MHT. Topical estrogen was more closely associated with reduced odds of soft‐tissue shoulder disorders compared with oral estrogen and oral combined formulations.

**Level of Evidence:**

Level III, therapeutic retrospective comparative study.

## Graphical abstract

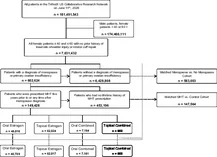

More than 2 million women in the United States and 50 million women worldwide enter menopause each year, and approximately 62% of these women experience joint and muscle pain.^1^, ^2^, ^3^ Despite the increasing number of women entering menopause each year, rates of menopausal hormone therapy (MHT) usage have declined from 26.9% in 1999 to 4.7% in 2020.^4^ This may be, in part, because of concern about complications including blood clots, stroke, or endometrial/breast cancer. Menopause is characterized by a decline in ovarian function and the production of female sex hormones. Estrogen is a key contributor to maintaining bone density in female patients, with the prevalence of osteoporosis increasing drastically during menopause. Estrogen deficiency has been shown to increase the sensitivity of musculoskeletal tissues to mechanical and metabolic stressors. Furthermore, postmenopausal women have been shown to experience reductions in muscle mass, muscle quality, and muscle strength and display increased markers of muscle proteolysis and neuromuscular degradation.^1^, ^5^, ^6^, ^7^, ^8^, ^9^

Women are 1.5 times more likely to have calcific deposits in the shoulder, twice as likely to have adhesive capsulitis (AC), and twice as likely to experience rotator cuff disease compared with men.^10^, ^11^, ^12^, ^13^, ^14^ Studies have also shown that female patients are more likely to suffer from shoulder impingement syndrome (SIS) and bicipital tendinitis (BT).^15^, ^16^, ^17^ Notably, the incidence of musculoskeletal pathology in women increases between the ages of 40 and 60, which coincides with the onset of menopause.^12^, ^18^ Of patients presenting with AC, 70% of them are women with the incidence peaking in the mid‐50s to 60s.^1^, ^19^

Given the detrimental effects of sex hormone loss, MHT has been highlighted as a method for addressing symptoms of menopause, including the associated musculoskeletal manifestations.^1^ MHT has robust efficacy in female patients who are recently menopausal and under the age of 60 to prevent fractures.^20^, ^21^ Some evidence has suggested that MHT may help maintain and increase muscle mass and strength, which can reduce fragility in postmenopausal women.^22^, ^23^ Previous research suggests that both oral and transdermal MHT have positive effects on bone mineral density.^24^ Thus, we postulate that MHT, given its role in stabilizing estrogen and progesterone levels, may be able to modulate musculoskeletal degradation, specifically in the soft tissue.

Despite the widespread prevalence of musculoskeletal complaints in perimenopausal women, there is limited research on the impact of MHT on soft‐tissue disorders of the shoulder. The purposes of this study were to determine whether perimenopausal women receiving MHT have decreased odds of developing soft‐tissue shoulder disorders compared with women without an active MHT prescription, specifically AC, bursitis, BT, calcific tendinitis (CT), atraumatic rotator cuff tear/rupture (RCTR), and SIS, and to quantify the impact of differing formulations and administration route. We hypothesize that MHT will be protective against developing soft‐tissue disorders of the shoulder due of the stabilizing effects of estrogen on menopausal symptoms and that the formulation and route of administration will significantly influence the MHT performance in this regard.

## METHODS

### Data Source

The TriNetX US Collaborative Research Network, a federated platform using electronic health record data from over 170 health care organizations with over 180 million patients, was used to identify subjects. This database contains deidentified electronic health records that are updated in real time, including patient demographics, diagnoses, medications, laboratory values, genomic information, and procedures. Institutional Review Board approval was not needed in this study because all data was deidentified per the standard defined in Section §164.514(a) of the Health Insurance Portability and Accountability Act Privacy Rule and was attested to by a qualified expert as defined in Section §164.514(b)(1) of the HIPAA Privacy Rule.

### Patient Selection

The database was queried on June 11, 2026. A preliminary analysis was conducted to establish menopause as a risk factor for shoulder soft‐tissue disorders. Patients were included in the menopause cohort if they were biological women between the ages of 40 and 60 years old with a diagnosis of menopausal and perimenopausal disorders (ICD‐10 N95.1) or primary ovarian insufficiency (ICD‐10 E28.3). Patients were included in the nonmenopause cohort if there was no diagnosis of menopause or primary ovarian insufficiency. Patients were excluded from all cohorts if they had a prior history of rotator cuff repair or antecedent shoulder trauma to minimize confounding by pre‐existing structural shoulder pathology (Supporting Information).

Systemic MHT was identified using standardized medication classes including estradiol, conjugated estrogens, and combined estrogen‐progestogen preparations (Supporting Information). Oral and topical formulations (for example transdermal patch) were included, whereas vaginal, injectable, and unknown preparations were excluded as a proxy for the inclusion of only systemic MHTs.

Within the base menopause population, five separate exposure versus comparator groups were established: any MHT, oral estrogen only, topical estrogen only, oral combined formulations, and topical combined formulations (Supporting Information). Only patients whose exposure instances were within a 5‐year window or after the recorded menopause diagnosis were included.

To establish the relation between a menopausal state and soft‐tissue disorders independent of MHT, a separate propensity score‐matched analysis was conducted. Patients were one‐to‐one propensity‐matched using the nearest matching neighbor algorithm on age, race, ethnicity, and medical risk factors including type 2 diabetes mellitus, hypothyroidism, hyperlipidemia, obesity, tobacco use, and chronic kidney disease because these have been previously independently associated with increased risk and represent a spectrum of metabolic comorbidities.^25^, ^26^, ^27^ Outcome ascertainment windows began 1 year after the index date, allowing for at least 1 year between initiation of MHT and any outcome inclusion.

Sensitivity analysis was also conducted as a check on cohort logic. The “Any MHT” cohort was analyzed against the control cohort without the 5 years prior or any time after temporal rule. Thus, the concurrent presence of the qualifying menopause diagnosis and MHT use was required.

### Outcomes

The primary outcomes were incidence of shoulder soft‐tissue disorders, which were assessed using their respective International Classification of Diseases‐10 codes. Soft‐tissue disorders included AC, BT, bursitis, CT, atraumatic RCTR, and SIS (Supporting Information).

### Statistical Analysis

All statistical analyses were performed on the TriNetX platform. One‐to‐one propensity score matching used a greedy nearest‐neighbor matching algorithm without replacement. The caliper distance was 0.10 standard deviations, and cohorts were randomly shuffled before matching. Patients were matched on age, race, ethnicity, type 2 diabetes mellitus, hypothyroidism, hyperlipidemia, obesity, tobacco use, and chronic kidney disease. Patients were considered well‐matched if the standardized mean difference was less than 0.1. The TriNetX platform was used to assess the dichotomous postmatch outcomes using an odds ratio (OR) with a 95% confidence interval (CI) to compare the number of patients who develop soft‐tissue pathology of the shoulder in patients prescribed MHTs compared with control cohorts.

## RESULTS

### Patient Selection

At the time of our search, there were 181,491,543 patients in the TriNetX US Collaborative Research Network (Figure 1). After applying the exclusion criteria, 7,031,432 patients remained. There were 602,624 patients in the menopause cohort and 6,428,808 patients in the nonmenopause cohort prior to matching. After matching, there were 583,003 patients in each cohort. Prior to propensity matching, there were 149,428 patients in the MHT cohort and 453,196 in the control cohort. After propensity score matching, there were 147,564 patients remaining. In the subgroup analyses before matching, there were 46,816 patients in the oral estrogen cohort, 62,824 patients in the topical estrogen cohort, 7164 patients in the oral combined cohort, and 902 patients in the topical combined cohort. In the subgroup analysis after matching, there were 46,789 patients in the oral estrogen cohort, 62,817 patients in the topical estrogen cohort, 7161 patients in the oral combined cohort, and 902 patients in the topical combined cohort.

**FIGURE 1:**
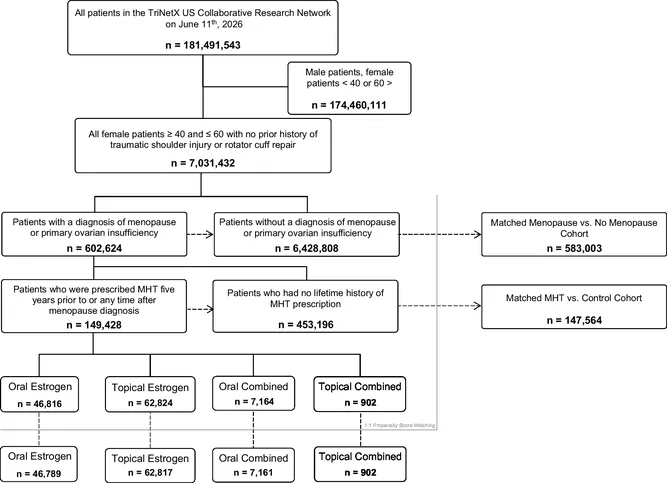
Strengthening the reporting of observational studies in epidemiology diagram of patient selection milestones for each cohort. (MHT, menopausal hormone therapy.)

### Patient Characteristics

After matching, there were no statistically significant differences between the patients in the MHT and non‐MHT cohorts (standardized mean difference < 0.1) (Table 1). The average age of the MHT cohort was 49.1 ± 5.4, and the non‐MHT cohort was 49.1 ± 5.3. The MHT cohort was 74.5% White, 9% African American, and 4.1% Asian, whereas the non‐MHT cohort was 74.6% White, 9% African American, and 4.1% Asian. There were no significant differences in any medical comorbidities after matching. Among all subgroup and sensitivity analyses, there were no significant differences after matching (Tables 2, 3, 4, 5, 6, 7).

**TABLE 1:** MHT and Control Patient Characteristics After 1:1 Propensity Score Matching

Variable	MHT Mean ± SD/n (%)	Control Mean ± SD/n (%)	SMD (Standard Difference)
Age at index	49.1 ± 5.4	49.1 ± 5.3	0.001
White	109,986 (74.5%)	110,036 (74.6%)	0.001
Unknown ethnicity	38,516 (26.1%)	38,471 (26.1%)	0.001
Hispanic or Latino	8796 (6%)	8828 (6%)	0.001
Not Hispanic or Latino	100,252 (67.9%)	100,265 (67.9%)	<0.001
Black or African American	13,220 (9%)	13,246 (9%)	0.001
Asian	6093 (4.1%)	6084 (4.1%)	<0.001
Hyperlipidemia, unspecified	26,086 (17.7%)	26,102 (17.7%)	<0.001
Other hypothyroidism	22,126 (15%)	21,961 (14.9%)	0.003
Type 2 diabetes mellitus	10,098 (6.8%)	10,138 (6.9%)	0.001
Overweight and obesity	35,578 (24.1%)	35,610 (24.1%)	0.001
Tobacco use	5079 (3.4%)	5052 (3.4%)	0.001
CKD	2381 (1.6%)	2230 (1.5%)	0.008

CKD, chronic kidney disease; MHT, menopausal hormone therapy; SD, standard deviation; SMD, standardized mean difference.

**TABLE 2:** Postmatching Demographics of Female Patients in the Menopause and No Menopause Diagnosis Cohorts

Variable	Menopause Dx, n (%)	No Menopause Dx, n (%)	Postmatch SMD
Age at index	48.3 ± 5.1	48.3 ± 5.2	0.011
White	386,816 (66.3%)	388,785 (66.7%)	0.007
Unknown ethnicity	166,718 (28.6%)	167,001 (28.6%)	0.001
Hispanic or Latino	39,306 (6.7%)	39,580 (6.8%)	0.002
Not Hispanic or Latino	376,979 (64.7%)	376,422 (64.6%)	0.002
Black or African American	70,998 (12.2%)	70,235 (12%)	0.004
Asian	26,417 (4.5%)	26,019 (4.5%)	0.003
Hyperlipidemia, unspecified	86,366 (14.8%)	83,851 (14.4%)	0.012
Other hypothyroidism	66,917 (11.5%)	67,492 (11.6%)	0.003
Type 2 diabetes mellitus	42,269 (7.3%)	45,496 (7.8%)	0.021
Overweight and obesity	125,311 (21.5%)	124,315 (21.3%)	0.004
Tobacco use	19,959 (3.4%)	20,865 (3.6%)	0.008
CKD	8520 (1.5%)	9272 (1.6%)	0.011

CKD, chronic kidney disease; Dx, diagnosis; SMD, standardized mean difference.

**TABLE 3:** Postmatching Demographics of Female Patients in the MHT and No MHT Cohorts: A Sensitivity Analysis

Variable	MHT Mean ± SD/n (%)	Control Mean ± SD/n (%)	SMD (Standard Difference)
Age at index	49.0 ± 5.3	49.0 ± 5.3	0.001
White	114,659 (74.3%)	114,709 (74.3%)	0.001
Unknown ethnicity	39,614 (25.7%)	39,581 (25.6%)	<0.001
Hispanic or Latino	9461 (6.1%)	9451 (6.1%)	<0.001
Not Hispanic or Latino	105,303 (68.2%)	105,346 (68.2%)	0.001
Black or African American	14,376 (9.3%)	14,364 (9.3%)	<0.001
Asian	6408 (4.2%)	6426 (4.2%)	0.001
Hyperlipidemia, unspecified	27,502 (17.8%)	27,503 (17.8%)	<0.001
Other hypothyroidism	23,231 (15%)	23,053 (14.9%)	0.003
Type 2 diabetes mellitus	10,758 (7%)	10,757 (7%)	<0.001
Overweight and obesity	37,926 (24.6%)	37,867 (24.5%)	0.001
Tobacco use	5491 (3.6%)	5425 (3.5%)	0.002
CKD	2507 (1.6%)	2376 (1.5%)	0.007

CKD, chronic kidney disease; MHT, menopausal hormone therapy; SD, standard deviation; SMD, standardized mean difference.

**TABLE 4:** Postmatching Demographics of Female Patients in the Oral Estrogen and Control Cohorts

Variable	Oral Estrogen Mean ± SD/n (%)	Control Mean ± SD/n (%)	SMD (Standard Difference)
Age at index	48.8 ± 5.5	48.8 ± 5.4	0.005
White	33,299 (71.2%)	33,293 (71.2%)	<0.001
Unknown ethnicity	20,438 (43.7%)	20,442 (43.7%)	<0.001
Hispanic or Latino	2422 (5.2%)	2423 (5.2%)	<0.001
Not Hispanic or Latino	23,929 (51.1%)	23,924 (51.1%)	<0.001
Black or African American	4936 (10.5%)	4931 (10.5%)	<0.001
Asian	2191 (4.7%)	2204 (4.7%)	0.001
Hyperlipidemia, unspecified	7219 (15.4%)	7233 (15.5%)	0.001
Other hypothyroidism	6091 (13%)	6062 (13%)	0.002
Type 2 diabetes mellitus	3415 (7.3%)	3432 (7.3%)	0.001
Overweight and obesity	10,095 (21.6%)	10,133 (21.7%)	0.002
Tobacco use	2005 (4.3%)	1960 (4.2%)	0.005
CKD	810 (1.7%)	729 (1.6%)	0.014

CKD, chronic kidney disease; SD, standard deviation; SMD, standardized mean difference.

**TABLE 5:** Postmatching Demographics of Female Patients in the Topical Estrogen and Control Cohorts

Variable	Topical Estrogen Mean ± SD/n (%)	Control Mean ± SD/n (%)	SMD (Standard Difference)
Age at index	49.6 ± 5.1	49.6 ± 5.1	0.001
White	49,624 (79%)	49,672 (79.1%)	0.002
Unknown ethnicity	24,101 (38.4%)	24,050 (38.3%)	0.002
Hispanic or Latino	3641 (5.8%)	3614 (5.8%)	0.002
Not Hispanic or Latino	35,075 (55.8%)	35,153 (56%)	0.003
Black or African American	5575 (8.9%)	5585 (8.9%)	0.001
Asian	1905 (3%)	1880 (3%)	0.002
Hyperlipidemia, unspecified	11,188 (17.8%)	11,184 (17.8%)	<0.001
Other hypothyroidism	9221 (14.7%)	9187 (14.6%)	0.002
Type 2 diabetes mellitus	4548 (7.2%)	4539 (7.2%)	0.001
Overweight and obesity	15,974 (25.4%)	15,958 (25.4%)	0.001
Tobacco use	2529 (4%)	2457 (3.9%)	0.006
CKD	1053 (1.7%)	974 (1.6%)	0.010

CKD, chronic kidney disease; SD, standard deviation; SMD, standardized mean difference.

**TABLE 6:** Postmatching Demographics of Female Patients in the Oral Combined MHT and Control Cohorts

Variable	Combined Oral Mean ± SD/n (%)	Control Mean ± SD/n (%)	SMD (Standard Difference)
Age at index	45.1 ± 5.5	45.1 ± 5.5	0.002
White	5543 (77.4%)	5554 (77.6%)	0.004
Unknown ethnicity	2264 (31.6%)	2262 (31.6%)	0.001
Hispanic or Latino	647 (9%)	651 (9.1%)	0.002
Not Hispanic or Latino	4250 (59.3%)	4248 (59.3%)	0.001
Black or African American	659 (9.2%)	653 (9.1%)	0.003
Asian	235 (3.3%)	242 (3.4%)	0.005
Hyperlipidemia, unspecified	1082 (15.1%)	1071 (15%)	0.004
Other hypothyroidism	1035 (14.5%)	1020 (14.2%)	0.006
Type 2 diabetes mellitus	474 (6.6%)	467 (6.5%)	0.004
Overweight and obesity	1983 (27.7%)	1979 (27.6%)	0.001
Tobacco use	252 (3.5%)	250 (3.5%)	0.002
CKD	75 (1%)	66 (0.9%)	0.013

CKD, chronic kidney disease; MHT, menopausal hormone therapy; SD, standard deviation; SMD, standardized mean difference.

**TABLE 7:** Postmatching Demographics of Female Patients in the Topical Combined MHT and Control Cohorts

Variable	Topical Combined Mean ± SD/n (%)	Control Mean ± SD/n (%)	SMD (Standard Difference)
Age at index	44.7 ± 5.21	44.7 ± 5.2	<0.001
White	583 (64.6%)	586 (65.0%)	0.007
Unknown ethnicity	266 (29.5%)	266 (29.5%)	<0.001
Hispanic or Latino	130 (14.4%)	130 (14.4%)	<0.001
Not Hispanic or Latino	506 (56.1%)	506 (56.1%)	0.003
Black or African American	135 (15.0%)	134 (14.9%)	<0.001
Asian	42 (4.7%)	41 (4.5%)	0.005
Hyperlipidemia, unspecified	128 (14.2%)	131 (14.5%)	0.003
Other hypothyroidism	108 (12.0%)	106 (11.8%)	0.010
Type 2 diabetes mellitus	61 (6.8%)	60 (6.7%)	0.007
Overweight and obesity	251 (27.8%)	250 (27.7%)	0.004
Tobacco use	32 (3.5%)	31 (3.4%)	0.006
CKD	10 (1.1%)	10 (1.1%)	<0.001

CKD, chronic kidney disease; MHT, menopausal hormone therapy; SD, standard deviation; SMD, standardized mean difference.

### Outcomes

Menopausal status was associated with significantly increased odds of all 6 shoulder pathologies compared with age‐matched controls: AC (OR 1.367, 95% CI 1.296‐1.442; *P* < .001), bursitis of the shoulder (OR 1.298, 95% CI 1.225‐1.376; *P* < .001), BT (OR 1.258, 95% CI 1.170‐1.354; *P* < .001), CT (OR 1.149, 95% CI 1.062‐1.243; *P* = .001), SIS (OR 1.260, 95% CI 1.200‐1.324; *P* < .001), and RCTR (OR 1.109, 95% CI 1.055‐1.165; *P* < .001) (Table 8). In the primary analysis, women prescribed any form of systemic MHT showed significantly lower odds of developing AC (OR 0.875, 95% CI 0.786‐0.973; *P* = .014), BT (OR 0.775, 95% CI 0.669‐0.898; *P* = .001), CT (OR 0.829, 95% CI 0.706‐0.974; *P* = .023), SIS (OR 0.873, 95% CI 0.792‐0.962; *P* = .006), and RCTR (OR 0.892, 95% CI 0.804‐0.991; *P* = .032) (Table 9). In the sensitivity analysis relaxing the temporal window requirement, findings were directionally identical to the primary analysis, with significant reductions in odds of AC (OR 0.853, 95% CI 0.769‐0.947; *P* = .003), BT (OR 0.779, 95% CI 0.675‐0.899; *P* = .001), CT (OR 0.823, 95% CI 0.703‐0.965; *P* = .016), SIS (OR 0.865, 95% CI 0.786‐0.951; *P* = .003), and RCTR (OR 0.887, 95% CI 0.801‐0.982; *P* = .021) (Table 10). The consistency between the sensitivity analysis and primary analysis confirms that the primary findings are not an artifact of the temporal window and that the reduction in odds holds regardless of how the timing relation is operationalized.

**TABLE 8:** Incidence and Odds of Soft‐Tissue Disorders of the Shoulder in Patients With and Without Menopause

Outcome	Menopause Dx	No Menopause Dx	Odds Ratio (95% CI)	*P* Value
AC	3199 (0.55%)	2359 (0.40%)	1.367 (1.296, 1.442)	**<.001**
Bursitis of the shoulder	2589 (0.44%)	2010 (0.34%)	1.298 (1.225, 1.376)	**<.001**
BT	1627 (0.28%)	1299 (0.22%)	1.258 (1.17, 1.354)	**<.001**
CT	1336 (0.23%)	1167 (0.20%)	1.149 (1.062, 1.243)	**.001**
SIS	3603 (0.62%)	2887 (0.50%)	1.260 (1.2, 1.324)	**<.001**
RCTR, nontraumatic	3310 (0.57%)	3008 (0.52%)	1.109 (1.055, 1.165)	**<.001**

*Note*: Bold denotes statistical significance of *P* < .05.

AC, adhesive capsulitis; BT, bicipital tendinitis; CI, confidence interval; CT, calcific tendinitis; Dx, Diagnosis; RCTR, rotator cuff tear/rupture; SIS, shoulder impingement syndrome.

**TABLE 9:** Incidence and Odds of Soft‐Tissue Disorders of the Shoulder in MHT and Non‐MHT Users

Outcomes	MHT, n (%)	Control, n (%)	Odds Ratio (95% CI)	*P* Value
AC	636 (0.43%)	728 (0.49%)	0.875 (0.786, 0.973)	**.014**
Bursitis of the shoulder	556 (0.38%)	587 (0.40%)	0.949 (0.845, 1.066)	.378
BT	315 (0.21%)	407 (0.28%)	0.775 (0.669, 0.898)	**.001**
CT	270 (0.18%)	326 (0.22%)	0.829 (0.706, 0.974)	**.023**
SIS	756 (0.51%)	869 (0.59%)	0.873 (0.792, 0.962)	**.006**
RCTR, nontraumatic	667 (0.45%)	749 (0.51%)	0.892 (0.804, 0.991)	**.032**

*Note*: Bold denotes statistical significance of *P* < .05.

AC, Adhesive Capsulitis; BT, bicipital tendinitis; CI, confidence interval; CT, calcific tendinitis; MHT, menopausal hormone therapy; RCTR, rotator cuff tear/rupture; SIS, shoulder impingement syndrome.

**TABLE 10:** Incidence and Odds of Soft‐Tissue Disorders of the Shoulder in MHT and Non‐MHT Users: A Sensitivity Analysis

Outcome	MHT, n (%)	Control, n (%)	Odds Ratio (95% CI)	*P* Value
AC	663 (0.43%)	778 (0.50%)	0.853 (0.769, 0.947)	**.003**
Bursitis of the shoulder	582 (0.38%)	624 (0.40%)	0.935 (0.835, 1.047)	.242
BT	333 (0.22%)	428 (0.28%)	0.779 (0.675, 0.899)	**.001**
CT	278 (0.18%)	338 (0.22%)	0.823 (0.703, 0.965)	**.016**
SIS	793 (0.51%)	920 (0.60%)	0.865 (0.786, 0.951)	**.003**
RCTR, nontraumatic	701 (0.45%)	792 (0.51%)	0.887 (0.801, 0.982)	**.021**

*Note*: Bold denotes statistical significance of *P* < .05.

AC, adhesive capsulitis; BT, bicipital tendinitis; CI, confidence interval; CT, calcific tendinitis; MHT, menopausal hormone therapy; RCTR, rotator cuff tear/rupture; SIS, shoulder impingement syndrome.

In the oral estrogen comparison, women prescribed oral estrogen had significantly increased odds of developing bursitis (OR 1.729, 95% CI 1.417‐2.109; *P* < .001), SIS (OR 1.377, 95% CI 1.158‐1.637; *P* < .001), and RCTR (OR 1.325, 95% CI 1.117‐1.570; *P* < .001) (Table 11). In the topical estrogen comparison, women prescribed topical estrogen had a significantly decreased risk of developing AC (OR 0.717, 95% CI 0.600‐0.856; *P* < .001), bursitis of the shoulder (OR 0.717, 95% CI 0.586‐0.876; *P* = .001), BT (OR 0.715, 95% CI 0.566‐0.904; *P* = .005), SIS (OR 0.729, 95% CI 0.617‐0.861; *P* < .001), and RCTR (OR 0.738, 95% CI 0.623‐0.874; *P* < .001) (Table 12). In the oral combined comparison, women prescribed combined oral MHT formulations had no significant differences in the odds of developing any soft‐tissue pathologies of the shoulder (Table 13). No results were available for the topical combined MHT formulations because the corresponding patient count was fewer than 10, leading to censoring by TriNetX to preserve patient confidentiality.

**TABLE 11:** Incidence and Odds of Soft‐Tissue Disorders of the Shoulder in Oral Estrogen MHT Users Versus Controls

Outcome	Oral Estrogen, n (%)	Control, n (%)	Odds Ratio (95% CI)	*P* Value
AC	276 (0.59%)	249 (0.53%)	1.109 (0.934, 1.317)	.236
Bursitis of the shoulder	265 (0.57%)	154 (0.33%)	1.729 (1.417, 2.109)	**<.001**
BT	145 (0.31%)	115 (0.25%)	1.263 (0.989, 1.614)	.061
CT	110 (0.24%)	93 (0.20%)	1.184 (0.898, 1.561)	.231
SIS	306 (0.65%)	223 (0.48%)	1.377 (1.158, 1.637)	**<.001**
RCTR, nontraumatic	309 (0.66%)	234 (0.50%)	1.325 (1.117, 1.57)	**<.001**

*Note*: Bold denotes statistical significance of *P* < .05.

AC, adhesive capsulitis; BT, bicipital tendinitis; CI, confidence interval; CT, calcific tendinitis; MHT, menopausal hormone therapy; RCTR, rotator cuff tear/rupture; SIS, shoulder impingement syndrome.

**TABLE 12:** Incidence and Odds of Soft‐Tissue Disorders of the Shoulder in Topical Estrogen MHT Users Versus Control Groups

Outcome	Topical Estrogen, n (%)	Control, n (%)	Odds Ratio (95% CI)	*P* Value
AC	211 (0.34%)	295 (0.47%)	0.717 (0.6, 0.856)	**<.001**
Bursitis of the shoulder	164 (0.26%)	229 (0.36%)	0.717 (0.586, 0.876)	**.001**
BT	120 (0.19%)	168 (0.27%)	0.715 (0.566, 0.904)	**.005**
CT	97 (0.15%)	111 (0.18%)	0.875 (0.666, 1.149)	.337
SIS	238 (0.38%)	328 (0.52%)	0.729 (0.617, 0.861)	**<.001**
RCTR, nontraumatic	233 (0.37%)	316 (0.50%)	0.738 (0.623, 0.874)	**<.001**

*Note*: Bold denotes statistical significance of *P* < .05.

AC, adhesive capsulitis; BT, bicipital tendinitis; CI, confidence interval; CT, calcific tendinitis; MHT, menopausal hormone therapy; RCTR, rotator cuff tear/rupture; SIS, shoulder impingement syndrome.

**TABLE 13:** Incidence and Odds of Soft‐Tissue Disorders of the Shoulder in Oral Combined MHT Users Versus Controls

Outcome	Combined Oral, n (%)	Control, n (%)	Odds Ratio (95% CI)	*P* Value
AC	33 (0.46%)	45 (0.63%)	0.732 (0.466, 1.148)	.172
Bursitis of the shoulder	29 (0.40%)	33 (0.46%)	0.878 (0.533, 1.448)	.610
BT	13 (0.18%)	25 (0.35%)	0.521 (0.266, 1.018)	.052
CT	22 (0.31%)	21 (0.29%)	1.047 (0.575, 1.906)	.880
SIS	56 (0.78%)	39 (0.54%)	1.441 (0.956, 2.172)	.079
RCTR, nontraumatic	29 (0.40%)	32 (0.45%)	0.907 (0.548, 1.5)	.702

AC, adhesive capsulitis; BT, bicipital tendinitis; CI, confidence interval; CT, calcific tendinitis; MHT, menopausal hormone therapy; RCTR, rotator cuff tear/rupture; SIS, shoulder impingement syndrome.

## DISCUSSION

The most important finding of this study was that female patients in menopause had a significantly higher incidence of shoudler soft tissue diagnosis than age‐matched controls. Within the menopausal cohort, women who were prescribed MHT had a decreased likelihood of developing soft‐tissue disorders of the shoulder, specifically AC, BT, CT, RCTR, and SIS, compared with a similar cohort of women who were not prescribed MHT. This suggests that exogenous sex hormone replacement in menopause or during the menopausal transition may confer a protective effect on periarticular connective tissues in the shoulder, consistent with the growing body of literature.

### Menopause as an Independent Risk Factor for Shoulder Soft‐Tissue Pathologies

In this study, menopause was associated with a significantly increased odds of AC, bursitis, BT, CT, SIS, and RCTR. Estrogen receptors are expressed in tenocytes, synovial membranes, articular cartilage, and vascular cells of the shoulder, and their activation regulates collagen synthesis, extracellular matrix turnover, and local inflammation.^11^, ^28^, ^29^, ^30^, ^31^, ^32^ The estrogen‐deficient state of menopause is associated with reduced collagen turnover, elevated proinflammatory cytokines, and decreased muscle mass.^33^, ^34^, ^35^ This collectively creates a biological basis for heightened susceptibility to soft‐tissue pathologies such as AC, CT, SIS, and RCTR which have been previously associated with menopause and estrogen deficiency in women.^36^, ^37^, ^38^, ^39^ This study confirms these associations and finds additional associations with BT and bursitis.

### Impact of MHT on Shoulder Soft‐Tissue Disorders

MHT was associated with significantly decreased odds of AC, BT, CT, SIS, and RCTR in menopausal women. Prior biologic and clinical studies have shown similar associations. MHT has been shown to improve the repair of muscle damage and remodeling of connective tissue within the skeletal muscle after exercise in postmenopausal women by upregulating connective tissue protein synthesis which occurs in response to mechanical loading.^28^ Cook et al. measured differences in Achilles tendon structure and diameter between active and inactive women who were currently using MHT or had stopped 12 months prior to the study. They found that MHT mitigates Achilles tendon thickening and structural abnormalities in the active women group. This suggests a normalizing effect of MHT on tendon health in active women.^40^ Cowan et al., in a 2 × 2 randomized control trial of transdermal MHT or placebo and tendon‐specific exercises or sham exercises on greater trochanteric pain syndrome, found that MHT combined with any form of exercise and patient education was effective in reducing pain and improving function.^41^

Estradiol administration in postmenopausal women has been associated with a higher tendon collagen fractional synthesis rate and reduced tendon stiffness. Estradiol and activation of the estrogen‐related receptor G‐protein coupled estrogen receptor have been shown to exert antifibrotic effects in animal models and in vitro models; importantly, inhibition of these pathways reverses the protective antifibrotic effects which shows a direct mechanistic link between estrogen signaling and fibrosis in AC.^11^ In vitro human cell‐based studies found that estradiol induces a significant increase in type I collagen in human dermal fibroblasts, which comprises 95% of rotator cuff tendon tissue. This may indicate a direct stimulatory effect on extracellular matrix protein production, enhancing the structural integrity and mechanical properties of skeletal muscle and connective tissue.^29^

Animal studies have found that estradiol‐17β stimulation increases fibroblast proliferation in tendon‐derived rat cells which promotes type I collagen deposition during tendon healing, both of which are critical for optimal functional recovery and biomechanical strength.^30^, ^31^ Reinke et al., in a single‐center retrospective cohort study, found that female patients between 40 and 60 years using hormone therapy had lower odds of AC, although this did not reach statistical significance because of a small sample size.^42^ Our study confirms this association at scale. Lin et al., in a large database study, found that after controlling for hormone use, menopause was still independently associated with CT, suggesting that hormone use did not fully eliminate the risk.^39^ This study defined hormone use broadly, including those using Western hormonal medications, traditional Chinese medicine, or female nutritional supplements, which may explain the discrepancy between our findings.

### Route of Administration and Formulation‐Specific Analyses

Interestingly, this study found topical estrogen to be superior to oral estrogen and oral combined MHT formulations in modulating soft‐tissue disorders of the shoulder. Topical estrogen was associated with reduced odds of AC, bursitis, BT, SIS, and RCTR. In contrast, oral estrogen was associated with increased odds of bursitis, SIS, and RCTR. Oral estrogen undergoes first‐pass hepatic metabolism which increases C‐reactive protein, sex hormone binding globulin, and coagulation factors and decreases IGF‐1, whereas topical/transdermal formulations do not.^43^, ^44^ Women prescribed topical estrogens may also differ systematically from oral estrogen users with respect to health care use, socioeconomic status, treatment adherence, and clinical prescribing practices.

The combination of increased systemic inflammation and reduced bioavailability of anabolic hormones may explain the paradoxical increase in inflammatory shoulder pathologies like bursitis and SIS seen with oral estrogen. The combined oral estrogen‐progestin cohort showed no significant change for any of the shoulder outcomes. The absence of a protective signal likely reflects contributions from first‐pass metabolism and the contribution of progestins. This cohort was also substantially smaller with wider CIs, suggesting that this analysis may have been underpowered to detect significant differences. The mechanism underscoring these findings cannot be determined from the present data and warrants further investigation.

Further research is recommended to assess the mechanistic impact of estrogen on the soft tissue within the shoulder and to understand estrogen's role in the cause of shoulder pathologies. Furthermore, the longitudinal impact of MHT on disease progression or reversal should be investigated to understand if there is an opportunity for MHT's therapeutic role to be expanded.

### Limitations

This study has various limitations. This study relied on data from a large database of electronic health records which means there may be inconsistencies in coding practices, data filtering, and mapping processes. Furthermore, the platform is reliant on diagnostic codes and structured data, which can contribute to the under‐representation of noncoded conditions or symptoms. Additionally, it was not possible to account for anatomical factors, dysfunctional rotator cuff or scapular musculature, postural abnormalities, and compliance with or length of MHT usage. The incidence of shoulder soft‐tissue pathologies in our study was lower than that of the general population, which may be because of undercounting via TriNetX or the strict exclusion criterion used in this study. Lastly, this study could not assess the longitudinal impact of MHT to assess the progression to disease state over time and the impact of targeted MHT interventions in at‐risk populations.

## CONCLUSIONS

In peri‐ and postmenopausal women, MHT use was associated with decreased odds of soft‐tissue shoulder disorders compared with individuals not using MHT. Topical estrogen was more closely associated with reduced odds of soft‐tissue shoulder disorders compared with oral estrogen and oral combined formulations.

## SUPPORTING INFORMATION

Additional supporting information can be found online in the Supporting Information section.

## DISCLOSURES

The authors (I.K.S., A.K.S., R.J.B., C.A.D., N.N.D., J.G.C., A.L.C.) declare that they have no known competing financial interests or personal relationships that could have appeared to influence the work reported in this article.

## Supporting information

Supplementary Material
