# Supplementary material for: Menopausal Hormone Therapy Decreases the Odds of Developing Soft‐Tissue Disorders of the Shoulder in Perimenopausal Women
Source: Arthrosc Sports Med Rehabil. 2026 Nov 8:e70158. Online ahead of print. doi: 10.1002/ars2.70158 (PMC13745816; doi:10.1002/ars2.70158)
Supplement: Supplementary file 1 [file ARS2-9999-e70158-s001.pdf]

Appendix 1

Inclusion Criteria

Code	System	Description
N95.1	ICD-10-CM	Menopausal and female climacteric states
E28.39	ICD-10-CM	Other primary ovarian failure

Exclusion Criteria

CPT/ICD-10-CM Code	Description
29827	Arthroscopy, shoulder, surgical; with rotator cuff repair
23410	Repair of ruptured musculotendinous cuff (eg, rotator cuff) open; acute
23412	Repair of ruptured musculotendinous cuff (eg, rotator cuff) open; chronic
23420	Reconstruction of complete shoulder (rotator) cuff avulsion, chronic (includes acromioplasty)
S42	Fracture of shoulder and upper arm
S43	Dislocation and sprain of joints and ligaments of shoulder girdle
S44	Injury of nerves at shoulder and upper arm level
S45	Injury of blood vessels at shoulder and upper arm level
S46	Injury of muscle, fascia and tendon at shoulder and upper arm level
S47	Crushing injury of shoulder and upper arm
S48	Traumatic amputation of shoulder and upper arm
S49	Other and unspecified injuries of shoulder and upper arm

Outcome Definitions

ICD-10-CM Code	Abbreviation	Description
M75.0	AC	Adhesive capsulitis of shoulder
M75.1	RCTR	Rotator cuff tear or rupture, not specified as traumatic
M75.2	BT	Bicipital tendinitis
M75.3	CT	Calcific tendinitis of shoulder
M75.4	SIS	Impingement syndrome of shoulder
M75.5	-	Bursitis of shoulder

Propensity Score Matching Variables

Code	Variable	Type
-	Age at index	Continuous
-	Race (White, Black/African American, Asian)	Categorical
-	Ethnicity (Hispanic/Latino, Not Hispanic/Latino, Unknown)	Categorical
E78.5	Hyperlipidemia, unspecified	Comorbidity
E03	Other hypothyroidism	Comorbidity
E11	Type 2 diabetes mellitus	Comorbidity
E66	Overweight and obesity	Comorbidity
Z72.0	Tobacco use	Comorbidity
N18	Chronic kidney disease (CKD)	Comorbidity

MHT Exposure Codes

Code	System	Route	Description
G03AA	ATC	Oral or Topical	Progestogens and estrogens, fixed combinations
G03AB	ATC	Oral or Topical	Progestogens and estrogens, sequential preparations
RxNorm 4083	RxNorm	Oral or Topical	Estradiol
RxNorm 4099	RxNorm	Oral or Topical	Estrogens, conjugated (USP)
